# Altered cerebral blood flow velocity features in fibromyalgia patients in resting-state conditions

**DOI:** 10.1371/journal.pone.0180253

**Published:** 2017-07-12

**Authors:** Alejandro Rodríguez, José Tembl, Patricia Mesa-Gresa, Miguel Ángel Muñoz, Pedro Montoya, Beatriz Rey

**Affiliations:** 1 Departamento de Ingeniería Gráfica, Universitat Politècnica de València, Camino de Vera s/n, Valencia, Spain; 2 Departamento de Neurología, Hospital Universitari i Politècnic La Fe, Valencia, Spain; 3 Departamento Psicobiología, Facultad de Psicología, Universitat de València, Blasco Ibáñez 21, Valencia, Spain; 4 Departamento de Personalidad, Evaluación y Tratamientos psicológicos, Universidad de Granada, Granada, Spain; 5 IUNICS, Universitat Illes Balears, Palma de Mallorca, Spain; University of Würzburg, GERMANY

## Abstract

The aim of this study is to characterize in resting-state conditions the cerebral blood flow velocity (CBFV) signals of fibromyalgia patients. The anterior and middle cerebral arteries of both hemispheres from 15 women with fibromyalgia and 15 healthy women were monitored using Transcranial Doppler (TCD) during a 5-minute eyes-closed resting period. Several signal processing methods based on time, information theory, frequency and time-frequency analyses were used in order to extract different features to characterize the CBFV signals in the different vessels. Main results indicated that, in comparison with control subjects, fibromyalgia patients showed a higher complexity of the envelope CBFV and a different distribution of the power spectral density. In addition, it has been observed that complexity and spectral features show correlations with clinical pain parameters and emotional factors. The characterization features were used in a lineal model to discriminate between fibromyalgia patients and healthy controls, providing a high accuracy. These findings indicate that CBFV signals, specifically their complexity and spectral characteristics, contain information that may be relevant for the assessment of fibromyalgia patients in resting-state conditions.

## Introduction

Fibromyalgia syndrome (FMS) is a chronic disease [1] characterized by widespread musculoskeletal pain, an abnormal pain response from normally non-painful stimuli (allodynia) and an excessive sensitivity to painful stimuli (hyperalgesia) in many tender points. Symptoms derived from FMS include permanent fatigue, insomnia or non-refreshing sleep, stiffness, cognitive and emotional difficulties as depressive symptoms or anxiety response. Although etiology and pathophysiology of FMS is unknown, the central nervous sensitization and the pain-inhibiting mechanisms seem to be affected leading to augmented nociceptive processing [2,3].

There are several brain areas that are activated in response to painful stimuli, including the primary and secondary somatosensory cortices, the insula, the anterior cingulate and the thalamus, as well as prefrontal and parietal regions, which compose the neuromatrix of nociception [4–6]. In the case of chronic pain patients, a more pronounced activation of this neuromatrix is observed [7,8]. Furthermore, resting-state networks of chronic pain patients are altered in comparison with normal population [9–12].

One of the techniques that has been recently proposed to evaluate the dynamics of brain activation associated to painful stimuli is Transcranial Doppler (TCD) monitoring [13–15]. It is a non-invasive ultrasound diagnosis technique that analyzes the hemodynamical variations in the brain by measuring cerebral blood flow velocities (CBFV) in main cerebral vessels [16,17]. Although the spatial resolution of TCD monitoring is limited to the areas supplied by the vessels under study, its high temporal resolution makes the technique suitable to complement other techniques such as fMRI to evaluate brain function.

The aforementioned TCD studies have found that there are measurable variations in CBFV of anterior cerebral arteries (ACA) and middle cerebral arteries (MCA) in response to different kinds of painful stimuli, being these changes more pronounced in FMS patients than in general population [13–15]. Furthermore, there have also been complementary studies to assess the relationship of FMS with cognitive and attentional deficits using TCD [18,19]. However, up to our knowledge, the resting-state features of the CBFV of FMS patients have not been evaluated yet using TCD.

Studies with TCD in the psychophysiological field have commonly based their analysis on the evaluation of the temporal evolution of the mean CBFV (calculated from the so-called envelope velocity) [16,17,20], although there are specific studies that have proposed alternative analyses based on other factors, such as the frequency contents of the envelope signal [21]. Besides, there are recent studies that have evaluated the resting-state CBFV signal from the MCA [22] and the ACA [23,24] in general population, using not only the envelope velocity but also the raw TCD signal. The raw signal is the ultrasound signal that is received by the TCD monitoring system without any processing. It contains information from all the blood cells moving at different velocities [25]. On the other hand, the envelope signal is an already processed signal that represents the instantaneous CBFV of the fastest blood cells, which generate the highest Doppler shift.

The studies about resting-state categorization of CBFV in general population [22–24] have also proposed the use of features extracted from both the raw and the envelope signals using not only temporal analyses, but also frequency, time-frequency and information theory analyses. These features can contribute to a better characterization of the CBFV signals in different groups of people or different experimental conditions, complementing typical approaches based on analyzing the temporal evolution of the signals. Sejdić et al. [22] and Huang et al. [24] observed sex-based differences in parameters from the three additional domains both for the envelope signals and the raw CBFV signals from MCA and ACA vessels.

Although previous studies that have applied TCD to evaluate CBFV characteristics of FMS patients have been based on temporal analyses of the signals, the described methodology based on frequency, time-frequency and information theory features is worth to consider in order to assess the resting-state characteristics of CBFV of FMS patients.

As previously described, TCD has started to be applied for evaluating differences in cerebral hemodynamics between patients and controls during pain processing. The current work will advance in this kind of analysis by evaluating other kinds of signal processing and feature extraction methods that can help to differentiate between FMS patients and controls.

Specifically, the main goal of the present work is to characterize the CBFV signals from FMS population in resting-state conditions in comparison with healthy population. Taking into account studies with general population [22], we propose the application of different signal processing methods based on time, information theory, frequency and time-frequency analyses in order to extract different features that enable us to characterize the CBFV from the ACA and MCA, vessels that feed different areas of the pain neuromatrix.

Our first hypothesis is that there will be significant differences between FMS patients and the control group in frequency, time-frequency and information theory parameters obtained from the CBFV signal. This first hypothesis is supported by the evidence from previous studies that have applied this kind of analyses in general population and by the fact that other neuroimaging techniques have observed that resting-state networks of chronic pain patients show alterations in comparison with healthy subjects [10–12].

Furthermore, our second hypothesis is that we expect to find an association between these TCD parameters and psychological and clinical measures related to depression, anxiety and pain. Previous studies [26,27] have observed higher levels of depressiveness and anxiety in FMS patients in comparison with healthy controls, Besides, previous studies with other neuroimaging tools have also found correlations between altered physiological parameters and scores obtained from depression and anxiety questionnaires [12]. For these reasons, we expect to find relations between depression and anxiety measures and specific frequency, time-frequency and information theory parameters calculated from the CBFV signal.

## Materials and methods

### Participants

Fifteen females with FMS aged between 36 and 67 years (mean age 53.08 years; SD 7.64) and fifteen pain-free females aged between 33 and 53 years (mean age 45.60 years; SD 6.17) with comparable sociodemographic data were recruited for the study. Patients were recruited via the Valencian Fibromyalgia Association (AVAFI). Exclusion criteria included neurological disorders, major psychiatric diseases, and migraines during the day of the experimental session, strokes or inflammatory causes of pain. The control group was recruited by means of announcements in notice boards of universities and sport associations. The exclusion criteria for the control group were the same applied to patients, and the presence of any kind of chronic pain disorder. All participants were right-handed based on Edinburgh Handedness Inventory responses (FMS group: mean 19.3, SD 5.16; Control group: mean 18.3, SD 4.87). All the participants gave their informed written consent prior to their inclusion in the study. The study was approved by the ethics committees of the Universitat Politècnica de València and of the Hospital Universitari and Politècnic La Fe.

### Clinical assessment

In order to evaluate the mood, manual preference and the impact of pain on participants’ lives, they had to complete several questionnaires before the experimental session: several visual-analog scales for assessing daily and worst pain intensity during the last week, and also the pain laterality (referring to which body side, right versus left was perceived more painful), the State-Trait Anxiety Inventory questionnaire (STAI [28]), the Edinburgh Handedness Inventory test [29,30], the Beck Depression Inventory (BDI-II; Spanish adaptation by [31]) and the West Haven-Yale Multidimensional Pain Inventory (WHYMPI: [32]; Spanish adaptation by [33]).

An additional questionnaire had to be answered at the end of the experimental session: a reduced version of the resting state questionnaire (RQ) [34]. It consisted of 12 yes/no items about the participants’ experience during the resting period, to evaluate if they were able to remain thought-free during this period.

### Physiological recordings

In order to measure the cerebral hemodynamic of participants, a commercial transcranial Doppler sonography device (Multi-Dop T; DWL, Germany) was used. Blood flow velocities (in cm/s) in ACA and MCA from both hemispheres were simultaneously monitored using two 2-MHz probes. Each probe was capable of conducting the exploration at two different depths at the same time. The probes were placed in the temporal bone window and fixed using a headset provided with the Doppler Box. The insonation depth of each probe varied between 50 and 55 mm to register MCA blood flow velocity, and between 65 and 70 mm to monitor ACA blood flow velocity.

QL software (provided with the system) was used to register the signals from the Doppler Box and export them to text files for later analyses. The CBFV signals were stored with a sampling rate of 100 Hz while the raw signal was extracted as a binary file sampled at 7042 Hz.

Blood pressure was measured using a wrist blood pressure monitor (R3 Intellisense; Omrom Healthcare Co., Ltd., Kyoto, Japan). Mean arterial pressure was calculated from systolic and diastolic pressures.

### Experimental design

Before starting the experiment, participants had to fill out all the questionnaires described above except from the RQ questionnaire. They also received information about the experiment prior to data acquisition.

Thereafter, the Doppler sonography probes with the headset were placed on the participant’s head by an experienced neurologist.

Once the probes were correctly located, participants received instructions to remain awake, in silence and to avoid any thought during a 5-minute resting period. After this period, the participants had to complete the RQ questionnaire. Finally, they had to complete other tasks, which are not considered in this study.

Blood pressure was measured before and after the resting period.

### Data analysis

The envelope and raw signals obtained by Transcranial Doppler were analyzed offline using MATLAB R2015b (The Mathworks Inc., Natick, MA, USA) and its toolboxes with custom MATLAB scripts. Only the measurements with a signal quality enough to allow their analyses were considered.

The preprocessing of the envelope and raw signals was applied to the 5-minute data corresponding to the resting period. The first step was a detection and correction of outliers caused by artifacts. In the case of the envelope signals, this was followed by a linear low-pass filter (with a 10 Hz cut-off frequency) that removed the effect of higher frequency artifacts. Finally, in order to avoid the dependence with the incidence angle between the ultrasound probe and the artery, the envelope signal was normalized dividing it by its mean during the resting period and multiplying by 100, in a similar way to other studies [20]. On the other hand, the raw signal was divided by its maximum value during the resting period to restrict its range to normalized values between -1 and 1 for all the subjects.

Several parameters were calculated to characterize the hemodynamic signals from different perspectives: time, frequency, time-frequency and information theory [22,24]. The specific parameters are described below. All the parameters were obtained from the ACA and MCA signals, in both hemispheres and for envelope and raw signals.

Finally, heart rate was calculated from the intervals between the peaks of the normalized envelop signal, also known as inter-systolic intervals [35].

#### Statistical features

Five statistical features widely used were calculated to characterize the signals from each vessel. These statistical features included mean, standard deviation (StD), variance, kurtosis and skewness. They were calculated both to the signals before normalizing and after normalizing.

#### Information-theoretic features

The goal of information-theoretic analysis is to assess the complexity (or predictability) and the regularity of signals and it is often applied in the analysis of biomedical signals [22,36]. The parameters that have been obtained in the current study to evaluate complexity and regularity of CBFV signals are Lempel-Ziv complexity (LZC) [37–39], entropy rate (EntrRate) [40], and multiscale entropy (MSE) [41,42].

The LZC is a non-parametric feature that evaluates the randomness of finite sequences in a one-dimension signal [38]. It is related to the number of distinct substrings and the rate of their recurrence along the signal [39]. High values reflect a more complex signal.

The entropy rate measures the regularity by means of the evaluation of repetitive sequence of patterns in a signal [40]. Entropy rate varies between 0 (maximum randomness) and 1 (maximum regularity) [22].

Finally, the MSE method evaluates the regularity of complex signals by applying a consecutive coarse-graining process to the signal by averaging a successively increasing number of data points (scale or length) in non-overlapping windows [41]. The sample entropy is calculated for each coarse-grained signal [42]. In this case, an entropy of 0 represents maximum regularity.

#### Frequency features

The frequency features that were calculated included peak frequency (PeakFreq), centroid frequency (CentFreq), bandwidth (BW) [22,24,43] and spectral power in different frequency bands. Power Spectral Density (PSD) was calculated using the Welch method with a Hamming window of 1024 points and a 50% overlap.

The peak frequency and the centroid frequency are measures that can be used to evaluate spectral changes in the signals, while the BW is used to measure the spectral spread [43]. In addition, the PSD provides information on how power is distributed as a function of frequency.

The peak frequency, the centroid frequency and the BW were calculated from the preprocessed signal and also after applying a more restrictive low-pass filter (cut-off frequency of 0.6 Hz), in order to remove the effect on these parameters of the cardiac cycle.

On the other hand, the spectral power in frequency bands described in previous TCD studies [44] was calculated: low frequency band (LF: 0.04–0.15 Hz), high frequency band (HF: 0.15–0.4 Hz) and the ratio between the LF and HF components (LF/HF ratio).

Finally, an additional band (0.12–0.25 Hz) (B012-015) that has shown accentuated fluctuations in the brain metabolism of chronic pain patients in resting-state was also analyzed [10]. The spectral power in this band was calculated and normalized with respect to the spectral power in the band up to 1 Hz.

#### Time-frequency features

A time-frequency analysis based on the wavelet transform was made in order to examine how spectral components change over time. In this analysis, two parameters were calculated: the relative energy from the approximation coefficients (RWEa10) in different time-frequency bands and the wavelet entropy (Wentropy). The discrete Meyer wavelet was used to obtain a 10-level discrete wavelet decomposition [22,45].

#### Statistical analysis

In order to explore the differences between the FMS and the control group regarding the questionnaire data and the extracted features from the cerebral hemodynamic responses, several statistical data analyses were applied. All the analyses were conducted using SPSS 16.0 (SPSS Inc., Chicago, USA) with a 0.05 significance level.

An analysis of variance (ANOVA) was applied. The between-subject factor was the experimental group (FMS vs. control). Dependent variables included the extracted features from bilateral ACA and MCA, the data from questionnaires and the heart rate. Prior to applying the ANOVA, Kolmogorov-Smirnov and Levene tests were used to check the different dependent variables for normality and homoscedasticity, respectively. If a specific variable did not follow normality criteria, a non-parametric test was applied instead (Mann-Whitney U).

Spearman correlations were calculated between the variables from questionnaires and the CBFV features to analyze possible relations between pain-related variables (depressiveness, anxiety, pain severity and pain intensity) and cerebral blood flow. Furthermore, Pearson correlations were calculated between the information-theoretic features and the heart rate in order to evaluate the effects of heart rate in the complexity and the regularity parameters.

Finally, a Linear Discriminant Analysis (LDA) [46] was applied to evaluate if the features obtained from TCD CBFV signals are suitable to discriminate between fibromyalgia patients and controls. LDA finds the best linear discriminant function that separates the groups under study. After selecting the features that will be included in the classification model, the LDA model was evaluated by means of a leave-one-out cross-validation method. This feature selection and classification method was applied over three different data sets. The first one was composed by all the calculated features, both from ACA and MCA, the second data set was only composed by the ACA features and the third data set was composed by the MCA features.

## Results

### Clinical data

The clinical data are summarized in Table 1 (including number of participants taking different kinds of medication).

**Table 1:** Clinical data from the FMS group and the control group. Data are presented as mean values ± SD. Bold text indicates the clinical parameters that have shown significant differences between groups (using F or χ^2^).

	Control group (n = 15)	FMS group (n = 15)	F[1,29] or χ^2^	p	Partial η2
**Intensity/laterality pain**					
Maximum intensity	**3.27 ± 3.22**	**6.67 ± 2.1**	**11.77**	**0.002**	**0.296**
Minimum intensity	**0.93 ± 2.63**	**4 ± 2.2**	**11.97**	**0.002**	**0.300**
Medium intensity	**2.27 ± 2.84**	**5.73 ± 2.12**	**14.35**	**0.001**	**0.339**
Current intensity	**1.07 ± 1.98**	**5.4 ± 1.92**	**37.01**	**<0.001**	**0.569**
Right-lateralization pain	**2.13 ± 2.48**	**6.73 ± 2.1**	**30.3**	**<0.001**	**0.520**
Left-lateralization pain	**1.53 ± 1.92**	**5.73 ± 2.8**	**23.06**	**<0.001**	**0.452**
**Medication**					
Antidepressants (%)	0 (0)	6 (40)	7.5	0.006	
Analgesics/relaxants/NSAIDS(%)	1 (7)	10 (67)	11.63	0.001	
Anxiolytics (%)	0 (0)	8 (53)	10.91	0.001	
**STAI**					
STAI-state	**16.4 ± 6.84**	**26.7 ± 11.2**	**9.32**	**0.005**	**0.250**
STAI-trait	**14.9 ± 8.48**	**31.4 ± 12.0**	**19.09**	**<0.001**	**0.405**
**BDI**	**5.8 ± 5.41**	**21.67 ± 13.1**	**18.82**	**<0.001**	**0.402**
**WHYMPI**					
Perceived support	3.42 ± 2.00	2.62 ± 1.62	1.45	0.239	0.049
Negative mood	**1.45 ± 1.23**	**3.88 ± 1.25**	**29.0**	**<0.001**	**0.493**
Pain interference	**0.42 ± 0.69**	**3.53 ± 1.7**	**42.87**	**<0.001**	**0.593**
Activity interference	**0.98 ± 1.17**	**3.88 ± 1.64**	**31.24**	**<0.001**	**0.512**
Pain severity	**1.25 ± 1.14**	**3.83 ± 1.4**	**30.67**	**<0.001**	**0.506**
Self-control	4.13 ± 1.42	3.97 ± 1.61	0.090	0.766	0.003
Distracting responses	**3.72 ± 0.68**	**2.5 ± 1.72**	**6.21**	**0.019**	**0.187**
Solicitous responses	**2.85 ± 1.43**	**1.65 ± 1.46**	**4.97**	**0.034**	**0.156**
Punishing responses	**0.14 ± 0.53**	**1.47 ± 1.57**	**8.99**	**0.006**	**0.250**
Household chores	4.30 ± 0.84	3.62 ± 1.23	3.13	0.088	0.100
Outdoor work	2.76 ± 0.81	2.21 ± 1.45	1.62	0.214	0.055
Activities away from home	1.91 ± 1.59	1.13 ± 1.45	1.96	0.172	0.066
Social activities	3 ± 0.89	2.13 ± 1.67	3.14	0.087	0.101

Focusing on clinical questionnaires, the FMS group showed significantly higher levels of pain intensity than the control group in the Pain Intensity/Laterality questionnaire.

Regarding anxiety measures (STAI questionnaire), significant differences were observed for the STAI-state and STAI-trait, with higher levels of anxiety for the FMS group. Regarding depression (BDI questionnaire), significantly higher values were obtained in the FMS group for overall depression scores.

Finally, the results obtained for the different scales and subscales of the WHYMPI questionnaire showed statistically significant differences for sections I and II. Section I is related to “pain experience”, and significant effects were found in the following subscales: negative mood, pain interference, activity interference and pain severity. In Section II (“response by significant others”), significant differences were found in the following subscales: distracting responses, solicitous responses and punishing responses. FMS patients perceived a lower psychological and instrumental support and a higher negative support by significant others than the control group. The subscales from section III (daily activity) did not reveal any significant effect.

No significant differences were found between the mean arterial pressure before and after the resting period, neither for the control group nor for the FMS group.

Finally, the resting questionnaire responses did not show any behavior that was considered sufficient to exclude any participant from the experiment.

### Transcranial Doppler signals

Some vessels were discarded because the quality of the signal was not enough to allow their analysis, giving a total number of 25 subjects for L-MCA (12 FMS group), 25 subjects for R-MCA (12 FMS group), 23 subjects for L-ACA (11 FMS group) and 24 subjects for R-ACA (11 FMS group).

For the envelope signals, Tables 2–5 summarize the obtained results for the different calculated features in the envelope CBFV signals from the four considered arteries. There were significant differences in several information-theoretic, frequency and power spectral density features between the control and FMS groups. However, there were no significant differences in any statistical or time-frequency feature.

**Table 2:** A summary of statistical parameters (calculated before and after normalization), information-theoretic, frequency and time-frequency features extracted from L-ACA envelope CBFV signals. All the values with no indicated units are dimensionless. Data are presented as mean values ± standard deviation. Bold text indicates the features that have shown significant differences between groups.

		L-ACA				
		Control group (n = 12)	FMS group (n = 11)	F[1,22]	p	Partial η2
**Statistical** **(before)**	***Mean (cm/s)***	50.47±6.05	53.27±7.67	0.959	0.339	0.044
	***StD (cm/s)***	13.22±2.32	13.48±2.15	0.079	0.781	0.004
**Statistical** **(after)**	***StD***	26.18±3.23	25.38±2.17	0.543	0.469	0.025
**Inf-Theor**	***LZC***	**0.46±0.01**	**0.48±0.03**	**4.669**	**0.042**	**0.182**
	***EntrRate***	0.90±0.03	0.87±0.04	3.729	0.067	0.151
	***MSE_1***	0.18±0.02	0.21±0.04	2.752	0.112	0.116
	***MSE_5***	0.47±0.07	0.53±0.14	2.135	0.159	0.092
	***MSE_10***	**0.57±0.08**	**0.69±0.15**	**5.346**	**0.031**	**0.203**
	***MSE_15***	**0.73±0.10**	**0.84±0.15**	**4.661**	**0.043**	**0.182**
	***MSE_20***	0.89±0.14	0.96±0.10	1.667	0.211	0.211
**Freq.** **<0.6Hz**	***PeakFreq (Hz)***	0.03±0.04	0.05±0.05	0.407	0.530	0.019
	***CentFreq (Hz)***	**0.12±0.02**	**0.15±0.03**	**8.860**	**0.007**	**0.297**
	***BW (Hz)***	0.11±0.02	0.13±0.03	2.805	0.109	0.118
**Freq.** **<10Hz** **(Global)**	***PeakFreq (Hz)***	1.13±0.14	1.26±0.14	3.905	0.060	0.158
	***CentFreq (Hz)***	1.58±0.18	1.70±0.17	2.463	0.131	0.105
	***BW (Hz)***	1.23±0.12	1.21±0.14	0.153	0.700	0.007
	***LF (Hz)***	5.57±2.65	4.34±2.24	1.438	0.244	0.064
	***HF (Hz)***	2.59±2.33	3.24±2.39	0.441	0.514	0.021
	***LFHFRat (Hz)***	**3.15±1.59**	**1.62±0.71**	**8.655**	**0.008**	**0.292**
	***B012-025***^***a***^	0.21±0.16	0.24±0.08	41.00	0.134	0.321
**Time-Freq.**	***RWEa10***	98.40±0.28	98.45±0.40	0.145	0.707	0.007
	***Wentropy***	0.14±0.02	0.14±0.03	0.135	0.717	0.006

^**a**^ A Mann-Whitney test was performed (data presented correspond to the statistic U, p value and effect size r).

**Table 3:** A summary of statistical parameters (calculated before and after normalization), information-theoretic, frequency and time-frequency features extracted from R-ACA envelope CBFV signals. All the values with no indicated units are dimensionless. Data are presented as mean values ± standard deviation. Bold text indicates the features that have shown significant differences between groups.

		R-ACA				
		Control group (n = 13)	FMS group (n = 11)	F[1,23]	p	Partial η2
**Statistical** **(before)**	***Mean (cm/s)***	55.28±12.5	53.19±11.4	0.179	0.677	0.008
	***StD (cm/s)***	14.37±2.83	13.72±2.47	0.353	0.558	0.016
**Statistical** **(after)**	***StD***	26.22±3.52	26.05±2.50	0.018	0.894	0.001
**Inf-Theor**	***LZC***	0.48±0.03	0.49±0.03	0.798	0.381	0.035
	***EntrRate***	0.88±0.04	0.85±0.05	1.696	0.206	0.072
	***MSE_1***	0.21±0.05	0.22±0.06	0.407	0.530	0.018
	***MSE_5***	0.55±0.20	0.59±0.20	0.183	0.673	0.008
	***MSE_10***	0.67±0.21	0.73±0.23	0.499	0.487	0.022
	***MSE_15***	0.81±0.20	0.90±0.22	1.094	0.307	0.047
	***MSE_20***	0.96±0.18	1.01±0.16	0.445	0.512	0.020
**Freq.** **<0.6Hz**	***PeakFreq (Hz)***	0.04±0.04	0.02±0.00	1.707	0.205	0.072
	***CentFreq (Hz)***	0.13±0.03	0.15±0.03	2.804	0.108	0.113
	***BW (Hz)***	0.11±0.02	0.13±0.02	1.716	0.204	0.072
**Freq.** **<10Hz** **(Global)**	***PeakFreq (Hz)***	1.14±0.13	1.25±0.14	4.236	0.052	0.161
	***CentFreq (Hz)***	1.65±0.19	1.78±0.19	2.803	0.108	0.113
	***BW (Hz)***	1.30±0.14	1.30±0.17	0.002	0.965	<0.001
	***LF (Hz)***	6.29±3.49	4.51±2.17	2.143	0.157	0.089
	***HF (Hz)***	2.86±2.74	2.66±1.71	0.045	0.834	0.002
	***LFHFRat (Hz)***	2.95±1.49	1.96±0.99	3.518	0.074	0.138
	***B012-025***^***a***^	0.20±0.16	0.23±0.8	44.00	0.119	0.325
**Time-Freq.**	***RWEa10***	98.33±0.42	98.39±0.27	0.146	0.706	0.007
	***Wentropy***	0.15±0.03	0.14±0.02	0.077	0.784	0.003

^**a**^ A Mann-Whitney test was performed (data presented correspond to the statistic U, p value and effect size r).

**Table 4:** A summary of statistical parameters (calculated before and after normalization), information-theoretic, frequency and time-frequency features extracted from L-MCA envelope CBFV signals. All the values with no indicated units are dimensionless. Data are presented as mean values ± standard deviation. Bold text indicates the features that have shown significant differences between groups.

		L-MCA				
		Control group (n = 13)	FMS group (n = 12)	F[1,24]	p	Partial η2
**Statistical** **(before)**	***Mean (cm/s)***	70.42±10.1	68.45±13.0	0.181	0.674	0.008
	***StD (cm/s)***	17.65±2.70	16.36±2.13	1.747	0.199	0.071
**Statistical** **(after)**	***StD***	25.21±3.22	24.17±2.03	0.936	0.343	0.038
**Inf-Theor**	***LZC***	**0.46±0.02**	**0.48±0.02**	**5.134**	**0.033**	**0.182**
	***EntrRate***	0.90±0.05	0.87±0.05	2.383	0.136	0.094
	***MSE_1***	**0.17±0.03**	**0.20±0.03**	**4.937**	**0.036**	**0.177**
	***MSE_5***	0.43±0.11	0.49±0.11	2.022	0.168	0.081
	***MSE_10***	0.55±0.12	0.63±0.12	2.703	0.114	0.105
	***MSE_15***	0.71±0.12	0.79±0.11	3.181	0.088	0.121
	***MSE_20***	0.86±0.14	0.91±0.12	0.927	0.346	0.039
**Freq.** **<0.6Hz**	***PeakFreq (Hz)***	0.03±0.03	0.04±0.04	0.198	0.661	0.009
	***CentFreq (Hz)***	**0.12±0.03**	**0.16±0.04**	**6.458**	**0.018**	**0.219**
	***BW (Hz)***	0.11±0.02	0.12±0.02	2.666	0.116	0.104
**Freq.** **<10Hz** **(Global)**	***PeakFreq (Hz)***	**1.14±0.14**	**1.26±0.14**	**4.590**	**0.043**	**0.166**
	***CentFreq (Hz)***	1.60±0.19	1.73±0.14	3.692	0.067	0.138
	***BW (Hz)***	1.24±0.14	1.26±0.12	0.176	0.679	0.008
	***LF (Hz)***	**8.13±3.01**	**5.40±2.36**	**6.282**	**0.020**	**0.215**
	***HF (Hz)***	3.85±3.17	4.11±2.32	0.053	0.820	0.002
	***LFHFRat (Hz)***	**3.02±1.75**	**1.57±0.81**	**6.857**	**0.015**	**0.230**
	***B012-025***^***a***^	**0.19±0.17**	**0.25±0.08**	**35.00**	**0.019**	**0.47**
**Time-Freq.**	***RWEa10***	98.31±0.59	98.63±0.23	2.969	0.098	0.114
	***Wentropy***	0.15±0.04	0.12±0.02	2.732	0.112	0.106

^**a**^ A Mann-Whitney test was performed (data presented correspond to the statistic U, p value and effect size r).

**Table 5:** A summary of statistical parameters (calculated before and after normalization), information-theoretic, frequency and time-frequency features extracted from R-MCA envelope CBFV signals. All the values with no indicated units are dimensionless. Data are presented as mean values ± standard deviation. Bold text indicates the features that have shown significant differences between groups.

		R-MCA				
		Control group (n = 13)	FMS group (n = 12)	F[1,24]	p	Partial η2
**Statistical** **(before)**	***Mean (cm/s)***	66.43±11.5	66.29±11.1	0.001	0.974	<0.001
	***StD (cm/s)***	16.50±2.69	16.64±2.75	0.016	0.899	0.001
**Statistical** **(after)**	***StD***	25.08±3.49	25.22±2.35	0.016	0.902	0.001
**Inf-Theor**	***LZC***	0.46±0.03	0.46±0.02	0.350	0.560	0.015
	***EntrRate***	0.90±0.04	0.90±0.04	0.181	0.675	0.008
	***MSE_1***	0.18±0.03	0.18±0.02	0.295	0.592	0.013
	***MSE_5***	0.43±0.15	0.42±0.09	0.016	0.899	0.001
	***MSE_10***	0.54±0.15	0.55±0.10	0.107	0.747	0.005
	***MSE_15***	0.69±0.13	0.72±0.09	0.365	0.552	0.016
	***MSE_20***	0.86±0.16	0.88±0.12	0.065	0.801	0.003
**Freq.** **<0.6Hz**	***PeakFreq (Hz)***	0.03±0.03	0.03±0.01	0.543	0.469	0.023
	***CentFreq (Hz)***	0.12±0.03	0.15±0.04	3.835	0.062	0.143
	***BW (Hz)***	0.11±0.02	0.12±0.02	2.505	0.127	0.098
**Freq.** **<10Hz** **(Global)**	***PeakFreq (Hz)***	1.15±0.14	1.26±0.14	3.391	0.078	0.129
	***CentFreq (Hz)***	1.64±0.21	1.73±0.14	1.498	0.233	0.061
	***BW (Hz)***	1.27±0.16	1.26±0.13	0.040	0.842	0.002
	***LF (Hz)***	7.24±2.63	5.39±1.98	3.894	0.061	0.145
	***HF (Hz)***	3.57±3.16	4.14±2.77	0.223	0.642	0.010
	***LFHFRat (Hz)***	**3.10±1.99**	**1.71±1.06**	**4.594**	**0.043**	**0.166**
	***B012-025***^***a***^	0.19±0.17	0.25±0.09	48.00	0.110	0.326
**Time-Freq.**	***RWEa10***	98.39±0.44	98.51±0.30	0.625	0.437	0.026
	***Wentropy***	0.14±0.03	0.13±0.02	0.577	0.455	0.024

^**a**^ A Mann-Whitney test was performed (data presented correspond to the U statistic, p value and effect size r).

**Fig 1:**
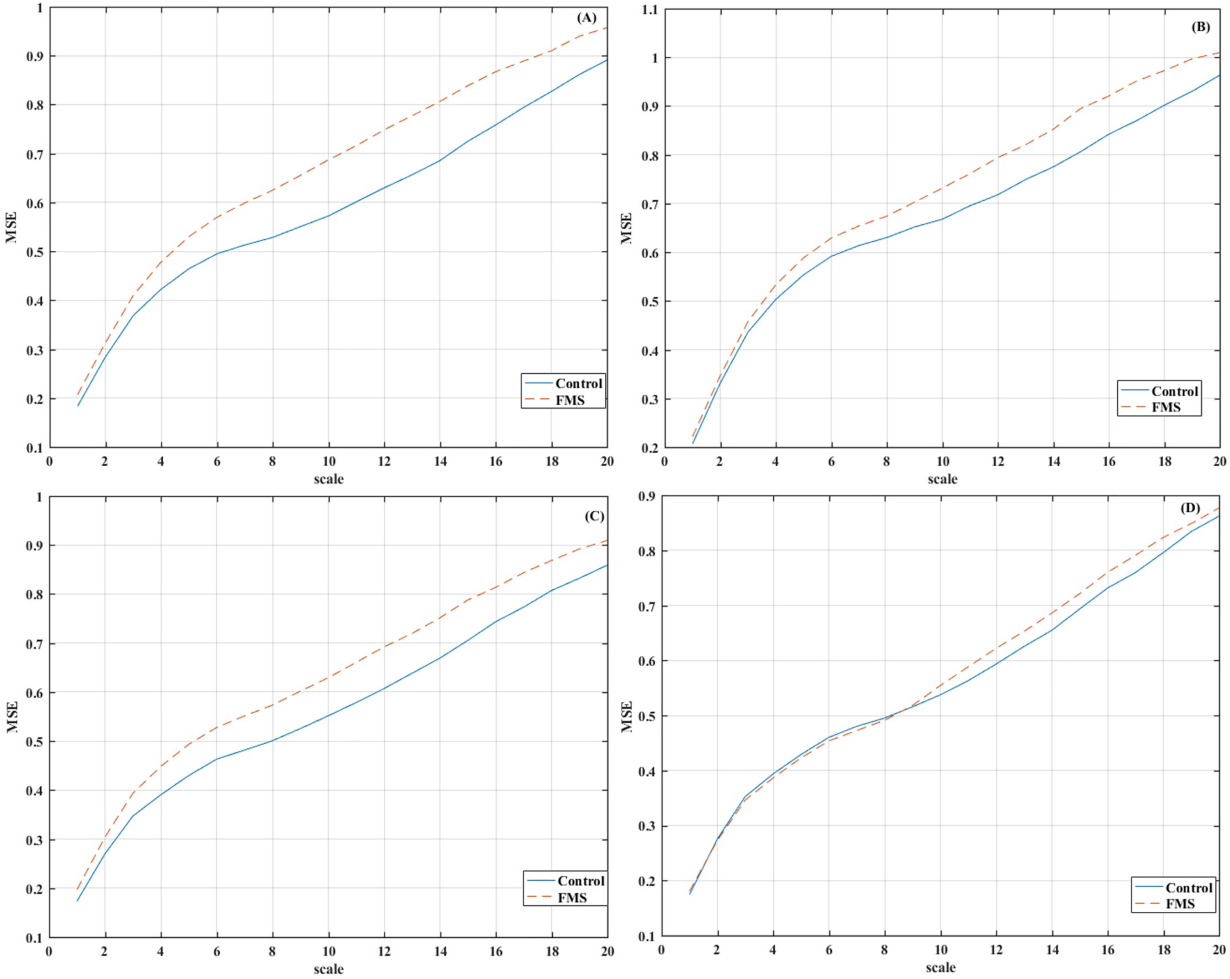
Multiscale entropy (MSE) analysis of the envelop signal time series in the following arteries: (a) L-ACA (b) R-ACA (c) L-MCA (d) R-MCA. Mean values (dimensionless) for each group (Control and FMS) are shown for scales 1 to 20.

The envelope signals from the FMS group in the left hemisphere were less predictable than those of the control group as measured by different information-theoric features. Mean MSE values for the different scales, arteries and groups can be observed in Fig 1.

Regarding the frequency analysis, results show a lower LF/HF ratio in different vessels of the FMS group and a higher spectral power in the frequency band between 0.12 Hz and 0.25 Hz in the L-MCA of this group. Besides, the centroid frequency in the lower frequencies spectrum (0–0.6 Hz) in the L-MCA and L-ACA and the peak frequency in the L-MCA were higher in the FMS group.

**Fig 2:**
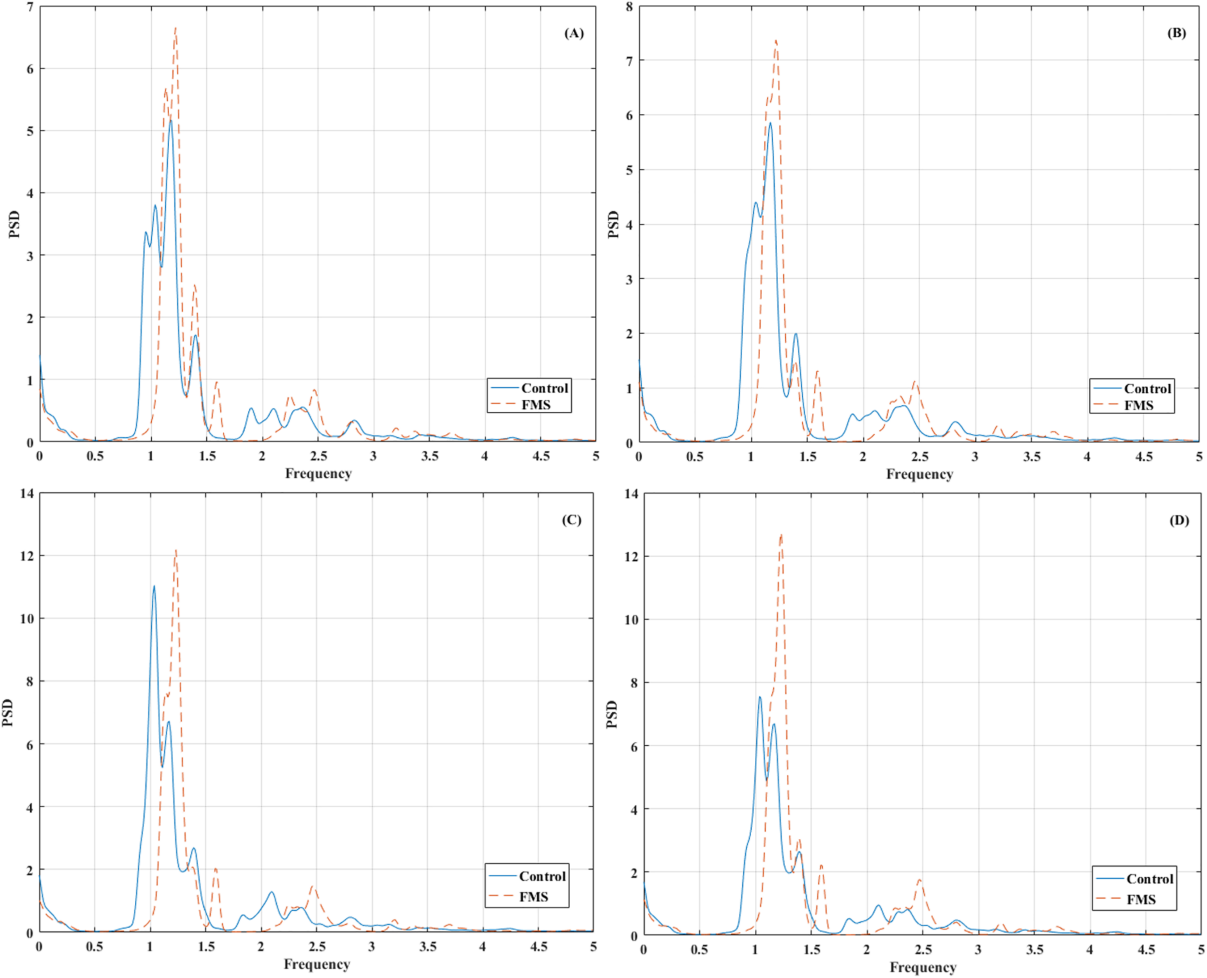
Grand average power spectral density (PSD) of the envelope cerebral blood flow velocity signal in the following arteries: (a) L-ACA (b) R-ACA (c) L-MCA (d) R-MCA. The frequency range between 0 and 5 Hz is included. Mean values (Hz^-1^) at each frequency bin for each group (Control and FMS) are shown.

**Fig 3:**
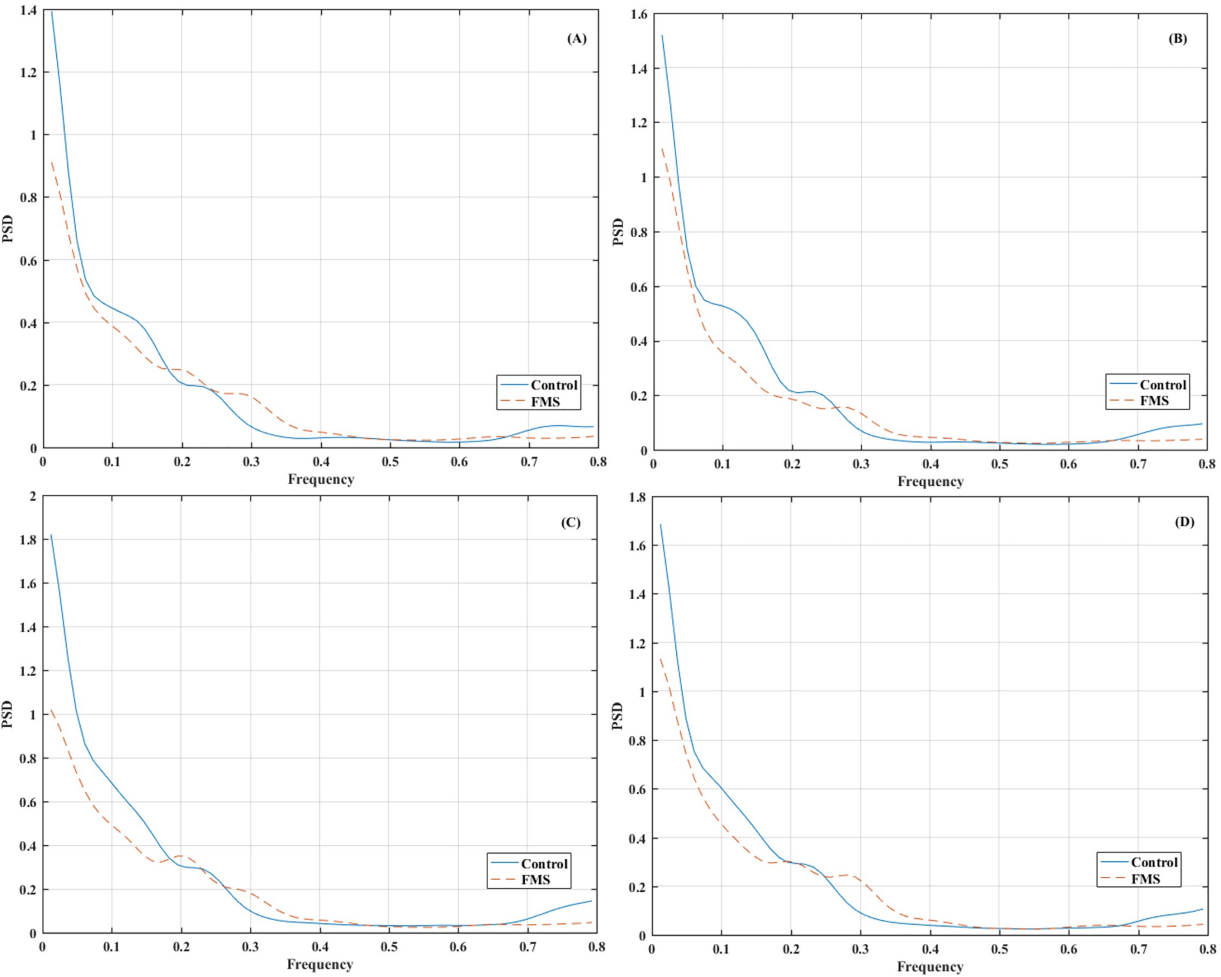
Grand average power spectral density (PSD) of the envelope cerebral blood flow velocity signal in the following arteries: (a) L-ACA (b) R-ACA (c) L-MCA (d) R-MCA. The frequency range between 0 and 0.8 Hz is included. Mean values (Hz^-1^) at each frequency bin for each group (Control and FMS) are shown.

The grand average power spectral density for each group in the different arteries can be observed in Fig 2 (including all the frequency range between 0 and 5 Hz–global spectrum) and in Fig 3 (focusing on a lower frequency range not affected by the cardiac cycle–lower frequencies spectrum).

The heart rate did not show any significant difference between the Control (68.568 ± 8.768) and the FMS (72.224 ± 11.37) groups (F(1,25) = 0.855; p = 0.364; partial η2 = 0.034).

Regarding raw data, no significant differences were found in any calculated parameter between the control group and the FMS group. The analyses described in following sections (correlation and LDA) were applied only to the envelope CBFV features.

### Correlation analysis

**Fig 4:**
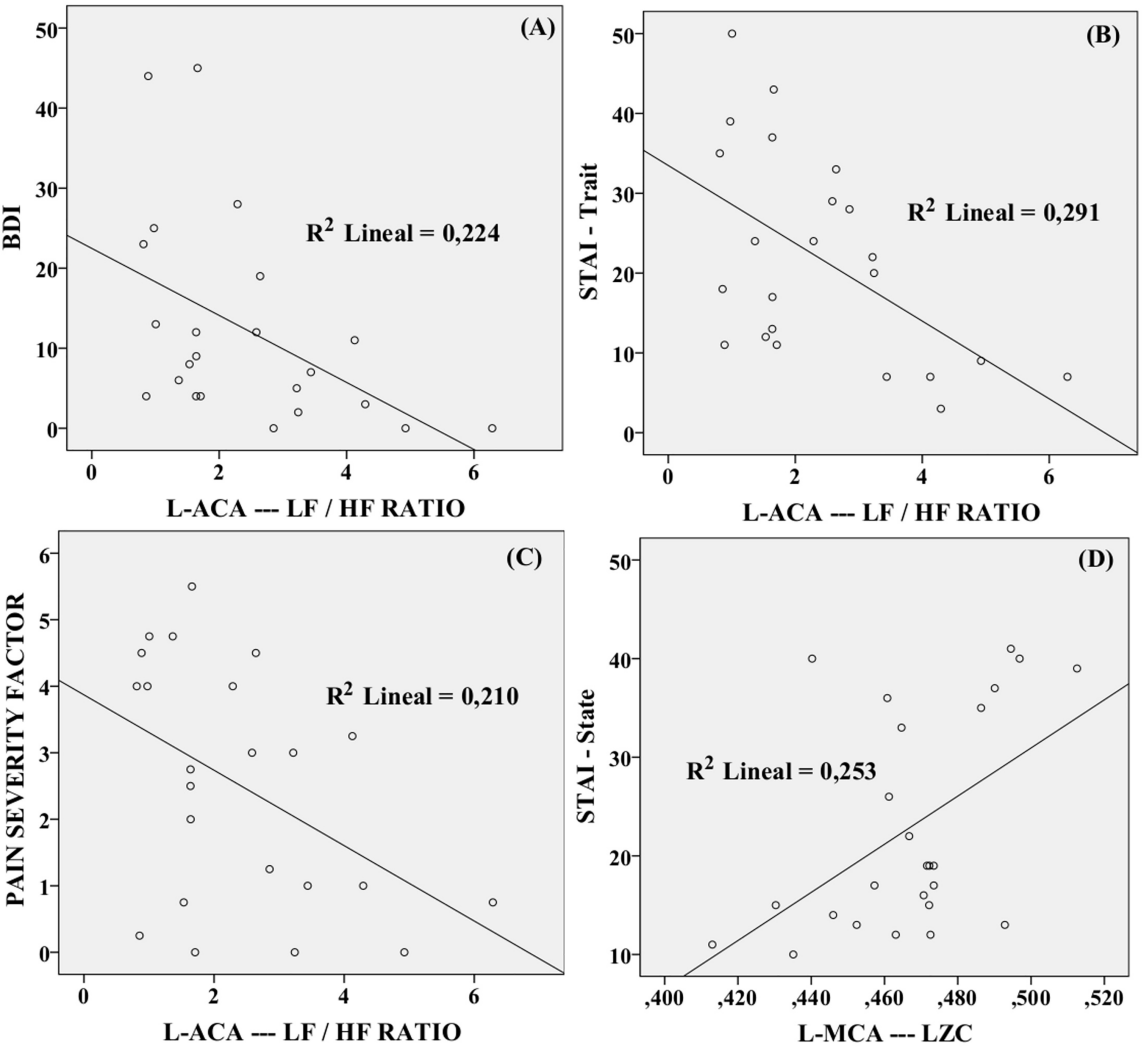
**Significant correlations between the LF/HF ratio from L-ACA and (a) BDI scores, (b) STAI-trait scores and (c) Pain Severity Factor, as well as, between (d) LZC from L-MCA and STAI-state.** No correlation was found between heart rate and information-theoretic parameters (p≥0.055).

Correlations were found between BDI questionnaire results, STAI-state, STAI-trait, pain severity factor (WHYMPI) and different envelope CBFV features. Significant correlations are shown in in Table 6. Some of the correlations are graphically represented in Fig 4.

**Table 6:** Correlations between BDI, STAI-state, STAI-trait and Pain Severity Factor (WHYMPI) and different parameters from the envelop BFV signal in the different vessels. Only the envelop BFV signal parameters that show significant correlations with each of the questionnaire values are included in the table.

		BDI	STAI-State	STAI-trait	Pain severity (WHYMPI)
**Inf-Theor**	***LZC***		**L-MCA**		
			r = 0.476		
			p = 0.016		
	***EntrRate***	**R-MCA**	**L-MCA**		
		r = -0.458	r = -0.450		
		p = 0.021	p = 0.024		
	***MSE (various scales)***		**L-ACA / L-MCA**		
			0.403 ≤ r ≤ 0.526		
			0.007 ≤ p ≤ 0.049		
**Frequency** **<0.6Hz**	***CentFreq***	**ACA / R-MCA**		**ACA**	**ACA**
		0.449 ≤ r ≤ 0.523		0.489 ≤ r ≤ 0.515	0.454 ≤ r ≤ 0.504
		0.009 ≤ p ≤ 0.024		0.012 ≤ p ≤ 0.015	0.014 ≤ p ≤ 0.026
	***BW***			**R-MCA**	
				r = 0.414	
				p = 0.040	
**Frequency** **<10Hz** **(Global)**	***PeakFreq***	**ACA**	**ACA / L-MCA**	**R-ACA**	
		0.419 ≤ r ≤ 0.451	0.430 ≤ r ≤ 0.467	r = 0.466	
		0.031 ≤ p ≤ 0.041	0.021 ≤ p ≤ 0.032	p = 0.022	
	***CentFreq***	**L-ACA / MCA**	**L-MCA**	**R-ACA**	
		0.446 ≤ r ≤ 0.564	r = 0.475	r = 0.439	
		0.005 ≤ p ≤ 0.025	p = 0.016	p = 0.032	
	***LF***				**L-ACA / L-MCA**
					-0.442 ≤ r ≤ -0.434
					0.030 ≤ p ≤ 0.035
	***HF***			**R-ACA**	
				r = 0.424	
				p = 0.039	
	***LFHFRatio***	**ACA / MCA**		**ACA / L-MCA**	**L-ACA**
		-0.535 ≤ r ≤ -0.435		-0.569≤ r ≤-0.399	r = -0.426
		0.008 ≤ p ≤ 0.030		0.004 ≤ p ≤ 0.048	p = 0.043
	***B012-025***		**R-ACA**	**R-ACA**	
			r = 0.427	r = 0.455	
			p = 0.037	p = 0.026	
**Time-Freq**	***RWEa10***	**L-MCA**		**L-MCA**	**L-MCA**
		r = 0.463		r = 0.423	r = 0.419
		p = 0.020		p = 0.035	p = 0.037
	***Wentropy***	**L-MCA**		**L-MCA**	**L-MCA**
		r = -0.425		r = -0.422	r = -0.402
		p = 0.034		p = 0.036	p = 0.046

### Linear discriminant analysis

In the case of the data set composed by all the features from the four vessels (excluding the statistical features, that had not shown any significant relevance in previous analyses), the selected features by the model were: LZC, LF, MSE factor 1 and the normalized power in the 0.12–0.25 Hz band for L-MCA; MSE factors 3 and 13 for R-ACA and MSE factor 14 for R-MCA. Initially, the classification model used all the features from all the samples as the training set to assess the LDA model. The classification achieved an accuracy of 91.30%. On the other hand, the classification results of the leave-one-out cross-validation (CV) approach achieved an accuracy of 100%.

Regarding the data set composed by ACA vessels features, the selected feature was the centroid frequency in the lower frequency band in the left hemisphere. The classification results using this feature from all the samples as the training set achieved an accuracy of 65.20% and the leave-one-out CV approach achieved an accuracy of 65.20%.

Finally, in the data set composed by MCA vessels features, the selected features were: LF/HF ratio and MSE1 for the left hemisphere and Entropy Rate and LF for the right hemisphere. The classification results using MCA vessels features from all the samples as the training set showed 91.7% of accuracy. The same accuracy was obtained with the leave-one-out CV approach.

The confusion matrices of the different classification models are shown in Table 7.

**Table 7:** Confusion matrices for LDA using all the features, ACA vessels features and MCA vessels features (except the statistical ones) from all the samples as the training set, using the leave-one-out cross validation approach, to discriminate between FMS group and control group.

	Predicted—All Features		Predicted-ACA Features		Predicted—MCA Features	
**Expected**	***Control***	***FMS***	***Control***	***FMS***	***Control***	***FMS***
***Control***	**11**	1	**9**	3	**11**	1
***FMS***	1	**10**	5	**6**	1	**11**
**CV**	***Control***	***FMS***	***Control***	***FMS***	***Control***	***FMS***
***Control***	**12**	0	**9**	3	**11**	1
***FMS***	0	**11**	5	**6**	1	**11**

## Discussion

This study has characterized the CBFV features obtained by TCD from FMS patients and healthy population. Results have shown that there exist significant differences in CBFV parameters of the TCD envelope signals of these groups in resting-state conditions.

The analyses of clinical pain measures showed results that are in line with previous studies [13]. More specifically, the FMS group had significantly higher levels of depressiveness and anxiety (state and trait) than the healthy population [1,47]. As expected, the FMS patients also showed significantly higher scores of pain intensity. These results are in accordance with those presented in other psychophysiological works where FMS patients participated [26,27].

Regarding the analyses of the registered TCD signals, we analyzed the envelope and raw CBFV signals, both for the FMS patients and the healthy population. Even though the raw CBFV analysis did not show any significant differences between the parameters from both groups, the envelope CBFV analysis showed differences in some of the parameters. The raw CBFV signal had been used previously as a valuable signal that can provide complementary information to the envelop CBFV analysis in resting-state conditions [22] and during the performance of cognitive tasks [48] in healthy population. However, in the present work only the envelope CBFV seems to include relevant information to characterize the groups under study.

Focusing on the envelope signal, the information-theoretic analysis showed significant differences for the LZC features in the two vessels of the left hemisphere. Although previous evidence indicates that several forms of pain are characterized by bilateral cerebral activations [49], there is not an exact correspondence between the activations and responses to pain that are observed in each hemisphere. Craggs et al. [50] used fMRI data from chronic pain and healthy control to estimate models of effective connectivity of pain-related processes. Although the models they obtained were similar between hemispheres, they observed group differences which involved the lack of paths of influence from S1 to S2 and from S1 to posterior insula in the left hemisphere of the FMS group (contralateral to the painful stimulation). Alterations of the connectivity between the default mode network (DMN) and different structures from each hemisphere have also been observed in FMS patients, also denoting a differentiation between hemispheres [51]. Patients have shown greater connectivity between the DMN and brain regions from the left hemisphere such as the left anterior, middle, and posterior insula and the left secondary somatosensory cortex [52]. The CBFV complexity features from the left hemisphere that are altered in the present study may be somehow related to these changes in connectivity that have been observed in previous studies in the same hemisphere.

LZC measures the possibilities of finding new patterns in a temporal series, so a larger LZC implies a more complex dynamical behavior [37]. Our results showed that the envelope signal of the FMS patients has a dynamical behavior with greater complexity and, hence, a lower predictability than the healthy participants. In this line, this analysis also revealed significant differences in the MSE for the scale factor 1 in L-MCA and for the scale factors 9 to 16 in L-ACA, being the envelope CBFV from the FMS patients more irregular and unpredictable than that from the control group.

The correlation results show an association of complexity measures such as LZC and MSE (scale factor 1) of the L-MCA with current anxiety (STAI State) and, in the case of LZC, with current pain level. Besides, the MSE values (scales factors 9 to 16) of the L-ACA are positively correlated with current anxiety (STAI State). The regularity (Entropy rate) of the CBFV signals in the R-MCA is negatively linked with the depression level (BDI measure).

Finally, the correlation analysis also shows the absence of relation between heart rate and information-theoretic parameters. Taking into account that the number of participants in the present study is rather small, this is a result that should be confirmed in future studies, as a larger sample size may be required to detect significant correlations between the studied parameters. However, it should be noted that this result is consistent with previous studies. Aboy et al. [38] had already concluded that the LZC was not influenced by the frequency of the signals being analyzed (in their study, simulated and intracranial pressure signals). Furthermore, previous TCD studies have observed that peripheral physiological mechanisms did not have any effect on slow changes of the cerebral blood flow velocity signal [35].

All these results seem to indicate that the complexity of the CBFV is influenced by different factors such as the depression, pain and anxiety levels of the participants in the study.

Although the bases of the observed differences in CBFV are unknown, there are evidences that have found a link between higher complexity in neuronal electrical activity and depression. Li et al. [53] described a significantly higher complexity in EEG from depression and schizophrenia patients in comparison with healthy participants during a resting task. They hypothesized that the origin of this increase in complexity could be in the activation of more neurons during this resting period in the case of patients. It has also been observed that depression patients had higher complexity in the MEG signal in resting-state conditions before starting a treatment than the healthy group [54]. Indeed, these complexity values decreased after an effective pharmacological treatment. This higher complexity in depression patients could be explained by a higher number of oscillatory systems working simultaneously and by increased frequency variability during the resting period in those patients. The authors of the study based their interpretation on previous works that had found that the EEG of depressed patients showed increased frequency variability caused by a higher disorganized neuronal activity [55] and that had concluded that the basis of the LZC complexity estimate is precisely the variability in the frequency components [38]. There are also studies that have got decreased complexity of these signals in major depressive patients in the context of other medical conditions [56]. Although we cannot assure that there is a direct relationship between the observations from neuronal activity and those from CBFV, it is interesting to consider those previous studies to analyze the origin of the higher CBFV complexity obtained in main cerebral arteries in the current work.

On the other hand, we have also found significant differences between the experimental groups in parameters from the frequency analysis. As can be observed in Fig 2, the CBFV spectrum is highly influenced by the cardiac cycle. A peak is present in all the arteries around 1.3 Hz (global peak frequency). Several harmonic peaks can be observed in higher frequency bands, with some concentrated power also present in lower frequency bands.

The global peak frequency is significantly higher in the FMS patients than in healthy participants (specifically, in L-MCA results). The CBFV component that has the biggest influence on the power spectrum is the cardiac cycle, which determines the position of the global peak frequency. Although the analyses with the current sample have not been able to detect significant differences between groups in the heart rate calculated from the intervals between the peaks of the normalized envelop signal, the higher values in the global peak frequency that have been obtained in the present study are coherent with the already known observation that FMS patients have higher heart rates than healthy population [26,57]. There is probably a different vegetative nervous response in both groups that can lead to these results [19].

When focusing on lower frequency bands, patients seem to have a decreased spectral power in the LF band in comparison with controls, and an increased spectral power in higher frequencies (Fig 3). The LF/HF ratio also presented significant differences for both hemispheres of MCA and for L-ACA, being the values of the healthy participants greater than those of FMS patients. The HF and LF fluctuations of CBFV have been described as secondary to the HF and LF fluctuations of the arterial blood pressure. The HF fluctuations of CBFV are secondary to HF fluctuations of arterial blood pressure (ABP) induced by respiration; LF fluctuations of CBFV are secondary to LF fluctuations of ABP, which originate from peripheral vasomotor activity and are additionally modified by cerebral autoregulation [44]. The LF/HF differences between FMS groups and healthy controls in resting state could reflect differences in peripheral vasomotor activity, vegetative nervous response, interference of drugs as well as subtle differences in cerebral autoregulation.

The correlation analyses show that the lower values the LF/HF ratio observed in the FMS group, for example in the L-MCA, are associated with higher values of both depression and anxiety (STAI trait). On the other hand, the LF spectral power of the L-MCA, which is also lower in the FMS group, is associated with pain indicators (Pain severity factor of the WHYMPI). Finally, the centroid frequency in the lower frequency band is associated with depression and anxiety (STAI state).

Our results also show that the fluctuations in the frequency band between 0.12 and 0.25 Hz are accentuated, in accordance to Malinen et al. [10], who had observed this effect in the BOLD fMRI signal in the insula of patients with chronic pain. This might be associated to an aberrant activity of the autonomic nervous system in the FMS patients. Modified vasomotion in the activated brain regions, typically occurring around 0.1 Hz, could be the origin of the difference between groups [58,59]. A posterior study was unable to find this behavior in the BOLD fMRI signal of FMS patients, but reported having found differences in even lower frequency bands [11]. Although further research will be needed to extract conclusions about these fluctuations in low frequency bands and the influence on them of processes such as cerebral autoregulation, baroreceptor reflex and vasomotion, TCD can be applied as a complementary tool to evaluate this kind of low frequency variations in the brain response.

All the obtained results are in accordance with previous studies that have demonstrated the relationship between negative emotional factors and activation of brain areas that are related to pain [60,61]. Therefore, our results would confirm that the complexity and the frequency features of CBFV are linked with other factors such as the depression levels, the state and trait anxiety, and pain indicators of the participants.

Finally, results from the LDA classification show that features extracted from CBFV could be useful to discriminate between FMS patients and healthy population by means of lineal models. The best accuracy was achieved when using all the calculated parameters from MCA and ACA, confirming the importance of characterizing the four arteries (MCA and ACA) in order to classify between FMS patients and healthy population. In addition, the stepwise features selection showed the high influence of the information-theoretic and frequency features in the LDA models, which would be in line with the previous results.

There are other studies with TCD that are applying classifiers to automatically detect cognitive states, i.e., [62–65]. Although their purpose is different from the one of the present study, it is interesting to indicate that their accuracies are in accordance with those obtained in the present work.

The present study has some limitations that should be taken into account. Firstly, the number of participants in this work is reduced. The results should be confirmed with a large population of different age ranges and ethnicities, making possible to analyze the influence on the results of other related factors such as medication. Due to this limitation, the correlation analyses between the TCD parameters and questionnaire scores have been conducted globally with the whole sample, and not separately for each group. This has to be taken into account when interpreting the correlation results. Most of the participants on the FMS group take prescription drugs, mainly analgesics, antidepressant and anxiolytic drugs. Therefore, the influence of drugs in our results cannot be completely discarded. Secondly, other physiological parameters were not continuously monitored during the procedure because the work did not have the goal of studying phenomena such as vasomotor activity or cerebral autoregulation. Consequently, we do not have direct indicators of the influence of those phenomena in the obtained results. Thirdly, the participants in this work were women. The results should be confirmed using also a sample with FMS male patients. Finally, the TCD technique has some limitations. It measures CBFV, which is just an indirect estimation of cerebral blood flow. Besides, its spatial resolution is rather low [17], being restricted to the cerebral areas supplied by the analyzed vessels. Consequently, it would not be possible to detect the activity in smaller foci of cerebral activation.

## Conclusion

The main goal of this study was to characterize the resting-state TCD in fibromyalgia patients. Therefore, we acquired raw and envelop CBFV signals in the main cerebral vessels (ACA and MCA). We observed that the fibromyalgia patients were characterized by a higher complexity of the envelope CBFV signal, as well as by a higher peak frequency, a higher 0.12–0.25 Hz band power, a lower LF/HF ratio and a higher centroid frequency in the lower frequency band in resting-state. In addition, these results showed a significant association with clinical pain parameters and emotional factors. Finally, we were able to classify with a high accuracy between FMS patients and healthy participants using the characterization features from the envelope CBFV signal.
